# Generic model for biological regulation

**DOI:** 10.12688/f1000research.110944.1

**Published:** 2022-04-13

**Authors:** Mauno Vihinen

**Affiliations:** 1 Department of Experimental Medical Science, Lund University, BMC B13, Lund, SE-221 84, Sweden

**Keywords:** poikilosis, biological regulation, lagom, therapy, disease, PLTR model

## Abstract

A substantial portion of molecules in an organism are involved in regulation of a wide spectrum of biological processes. Several models have been presented for various forms of biological regulation, including gene expression regulation and physiological regulation; however, a generic model is missing. Recently a new unifying theory in biology, poikilosis, was presented.  Poikilosis indicates that all systems display intrinsic heterogeneity, which is a normal state. The concept of poikilosis allowed development of a model for biological regulation applicable to all types of regulated systems. The perturbation-lagom-TATAR countermeasures-regulator (PLTR) model combines the effects of perturbation and lagom (allowed and sufficient extent of heterogeneity) in a system with tolerance, avoidance, repair, attenuation and resistance (TARAR) countermeasures, and possible regulators. There are three modes of regulation, two of which are lagom-related. In the first scenario, lagom is maintained, both intrinsic (passive) and active TARAR countermeasures can be involved. In the second mode, there is a shift from one lagom to another. In the third mode, reguland regulation, the regulated entity is the target of a regulatory shift, which is often irreversible or requires action of another regulator to return to original state. After the shift, the system enters to lagom maintenance mode, but at new lagom extent. The model is described and elaborated with examples and applications, including medicine and systems biology. Consequences of non-lagom extent of heterogeneity are introduced, along with a novel idea for therapy by reconstituting biological processes to lagom extent, even when the primary effect cannot be treated.

## Introduction

Poikilosis is a new unifying biological theory according to which inherent pervasive variation is a normal state for all biological systems ranging from subatomic particles to the biosphere ( Vihinen, 2020a). Traditionally, heterogeneity has been considered negative noise; however, it is an integral part of any system and has many consequences. Biological systems are not binary on-off switches, there are typically continua of states. Many systems balance between benefits and disadvantages of heterogeneity as variation can be tolerated, it can be harmful or beneficial.

Heterogeneity is generated both passively, e.g., by differential gene expression in cells of a tissue, and actively, such as the generation of the immense variation for immunological recognition molecules. Poikilosis facilitates biodiversity of species, populations and ecosystems within the biosphere; differences between cells, individuals and populations; differences at genetic, molecular, structural, physiological, interindividual and other levels; and thereby a large pool of possible responses to changes in conditions. The list of different forms of heterogeneity is endless.

Regulation is a common feature in biological systems. Examples include gene expression; signalling and metabolic pathways; enzyme and more generally protein activation and regulation, including allostery; developmental processes; effects of hormones and growth factors; cell cycle, cell fate and differentiation; regeneration; and growth control. Although the processes are very different, the principles of the regulation are the same in all these systems. There is a regulated entity, function, activity or other property that is controlled.

Consideration of poikilosis has already been shown to demand alterations to concepts and practices in science. New definitions have been presented for life, disease, death, senescence ( Vihinen, 2020a), experiments, measurements, their analysis and interpretation ( Vihinen, 2021b), health, disease and pathogenicity ( Vihinen, 2017, 2020b). Here, a new model and explanation is provided for biological regulation, applicable at any level of regulation, system and organism. The model is based on poikilosis, lagom, TARAR countermeasures (TCMs), regulators and emergent properties of complex systems. Examples are discussed to highlight the application of the new model of regulation.

### Poikilosis and lagom

Poikilosis is pervasive, but all variations and their extents are not compatible with biological processes and systems. Acceptable variation ranges are called *lagom* and defined as *suitable, sufficient, allowed and tolerated extent of variation at any level in an organism, population, biological system or process* ( Vihinen, 2020a) *.*

**Figure 1.**
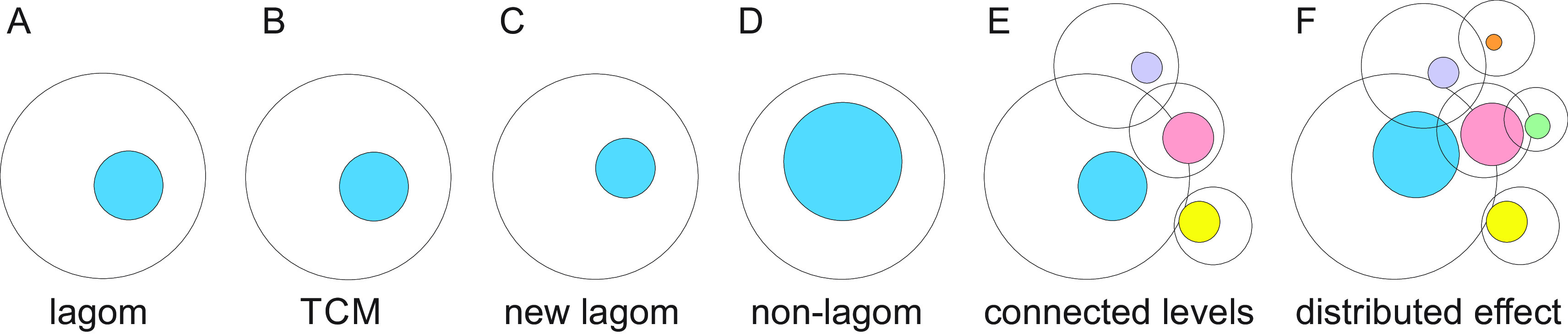
Description of lagom and non-lagom. A. The blue sphere indicates lagom and the white sphere the space of possible states. B. The effects of small or medium perturbation are restricted by TCMs thereby keeping the lagom extent of variation. C. Somewhat larger perturbation changes the extent and/or position of lagom. D. Larger perturbation causes the system to enter non-lagom state that is no more controlled by TCMs. E. Levels of molecules, systems etc. are highly connected. For each level the lagom extent is indicated as well as the possible range of heterogeneity. The colored spheres indicate lagom extents. F. Non-lagom extent in one level (blue) can affect some other levels and cause them to become non-lagom and extend to further levels. The effect on some levels is within the lagom extent and there is no change in the position or extent of lagom whereas others can enter non-lagom state.

Life does not strive towards perfection, rather at lagom (sufficient and relevant) reactions and responses ( Figure 1A). Enzymes are an example of lagom activity. Although the highest known increase in reaction rate is 10 ^26^-fold ( Edwards *et al*., 2012), most enzymes are much less efficient, but efficient enough for the organism. Another aspect of lagom in enzymes is that they are not entirely specific. Enzymes are promiscuous and may even have several activities ( Khersonsky *et al*., 2006; Vihinen 2021a). Similarly, other functions are tuned for lagom responses. The goal of life is survival of the individual and species. There is no selection pressure to increase activity or functionality of biological processes beyond lagom extent or to regulate a system beyond the lagom level pertinent to it.

In a system at lagom state there is a balance between heterogeneity producing and restricting processes. Lagom extent originates from the combination of intrinsic properties, active processes and emergent features. From the perspective of regulation, lagom is related to system level robustness and resilience against changes and perturbations.

Emergence is common in complex systems, as in biology, where emergent properties differ from the properties of the constituent components, such as molecules, processes, pathways etc. Life is an example of emergence in biology. Emergence is an explanation when behaviour of a complex system containing many interactions between system components cannot be attributed to the sum of the components. Emergent processes have been identified in a wide range of biological phenomena, such as quorum sensing in bacterial colonies ( Dalwadi and Pearce, 2021), robustness of biological processes ( Kitano, 2004; Masel and Siegal, 2009), evolution of biological flexibility ( Badyaev and Morrison, 2018), and synthetic biology ( Benner and Sismour, 2005).

TARAR is an abbreviation for tolerance, avoidance, repair, attenuation and resistance, mechanisms that can be intrinsic or adaptive. TCMs were originally discussed as effect-restricting mechanisms for protein variations ( Vihinen, 2021a); however, these mechanisms are generic and functional at all levels ( Figure 1B). Level means in here any molecule, process, or system and without any hierarchy or extent.

Once the extent of perturbation cannot be restricted, the system either enters to another lagom level with higher or lower extent of heterogeneity ( Figure 1C) or becomes non-lagom ( Vihinen, 2021b) ( Figure 1D). A system has a non-lagom condition when increased perturbations consistently increase values for the measurand, e.g., since return to lagom is not possible without external actions or changes in the environment. The consequences depend on the level and how it is linked to other levels, system-wide effects, and so on. Non-lagom variation can affect connected levels, and if it is large enough, even cause some of them to become non-lagom ( Figure 1E and F) and spread the effects to additional levels.

### TARAR countermeasures

**Figure 2.**
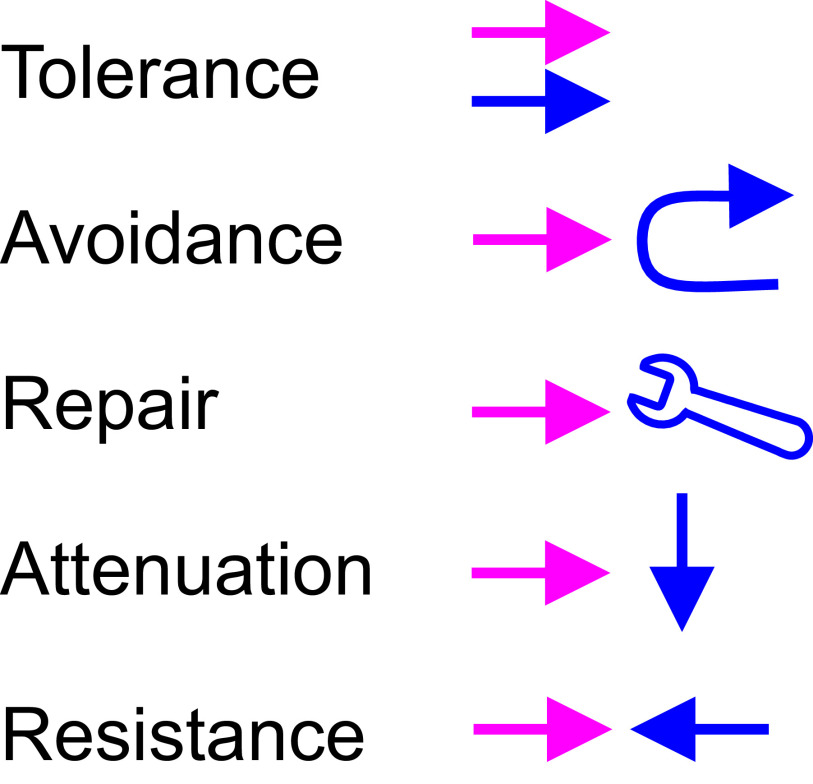
Graphical description of TARAR countermeasures. Perturbation is shown in red and TCMs in blue. The perturbation co-exists in tolerance. In avoidance the TCMs protect the system by preventing interaction with potentially harmful substances. Repair mechanisms renew or reconstitute the system, while attenuation processes reduce the effect of perturbation. Resistance mechanism oppose the effects of a perturbation.

TCMs are either active or systemic and they are inbuilt to processes, cells and organisms ( Figure 2). Variation tolerance is a general biological countermeasure. Many biological systems are tolerant for different perturbations. In the case of genetic variations, tens to hundreds of thousands of lesions appear daily in every cell ( Nakamura and Swenberg, 1999), almost all of which are corrected. Most of the remaining variations are harmless and tolerated and have no phenotypic effect, i.e., they do not lead to diseases or affect fitness or other properties. Disease tolerance was originally described in plants ( Politowski and Browning, 1978), then applied to animals ( Råberg *et al*., 2007), mainly in relation to immunology and subsequently extended to other diseases ( McCarville and Ayres, 2018; Medzhitov *et al*., 2012). In tolerance, the organism does not eradicate the disease-causing agent, instead reduces its impact. Tolerance may appear also because the intruding microbe, parasite or perturbation-causing agent or process cannot be eliminated. For an organism, tolerance of e.g., an infectious microbe can be beneficial, because of the smaller overall effect and damage than when the agent is eradicated.

In animals, avoidance due to distaste and disgust are the major ways to reduce risk of exposure for pathogen and parasite infections ( Curtis *et al*., 2011). There are several general strategies which are largely shared by various species including avoidance of pathogenic and parasitic organisms, controlled exposure to train the immune system, behaviours of sick individuals, selection of mating partners and helping sick individuals ( Hart, 1990). Disease avoidance here does not relate to stigmatization ( Oaten *et al*., 2011), instead, it means mechanisms to reduce the risk of infection.

Repair mechanisms reduce the effects of functional variants, such as dosage or genetic/genomic suppression ( Hodgkin, 2005; Prelich, 1999; van Leeuwen *et al*., 2017) and chaperone activities that refold misfolded proteins. Repair processes are active by nature and include, among others, DNA ( Chatterjee and Walker, 2017) and bone repair ( Salhotra *et al*., 2020), tissue repair and wound healing ( Gurtner *et al*., 2008). Regeneration is another renewal process. Repair mechanisms can be performed either by normal cellular and bodily activities and abundances of the molecules and other components or they are induced by a variant or perturbation and its effects.

Attenuation means robustness and resilience of the cells, systems, tissues, organisms or populations against perturbations by intrinsic and extrinsic cues. These buffering mechanisms are passive but have likely evolved with active processes and been selected during evolution ( de Visser *et al*., 2003). Cell population resistance has been shown to emerge from cellular heterogeneity ( Paszek *et al*., 2010).

Attenuation mechanisms restrict the extent of the phenotypic effects so that minor alterations are not phenotypically relevant as they are hidden among normal heterogeneity in the system. There is an extensive literature on robustness (canalization) ( Kitano, 2007; Waddington, 1942; Whitacre, 2012); however, the underlying mechanisms have not been fully elaborated. In poikilosis terminology, attenuation is closest to robustness. Redundancy (also called degeneracy) is a common form of attenuation. In yeast, at least one fourth of gene deletions without phenotypic effect are thought to be compensated by duplicate genes ( Gu *et al*., 2003).

Resistance mechanisms are both active and systemic and include mechanical, cellular, chemical, biochemical and immunological defence mechanisms. Resistance is a generic countermeasure against many perturbations. At organism level, epithelial cells form a mechanical protective barrier. Chemical and biochemical molecules which neutralize or defend against infectious agents. Numerous resistance mechanisms and processes exist for immunological but also for non-immunological perturbations. Some organisms have developed resistance to toxic compounds ( Ujvari *et al*., 2015), antibiotics ( MacGowan and Macnaughton, 2017) or other drugs ( Blasco *et al*., 2017). Some individuals even resist effects of complete (homozygous) gene knockouts ( MacArthur *et al.*, 2012; Narasimhan *et al*., 2016; Saleheen *et al*., 2017), apparently due to resistance and attenuation mechanisms ( Vihinen, 2021a).

Countermeasures are a combination of several intrinsic and adaptive measures, processes and reactions. Adaptive countermeasures, analogous to adaptive immunity, are mounted in response to alterations and vary depending on the type, location, timing etc. of the effective perturbation.

### Variation zone, lagom extent and level interactions

Variation zone is a reconstruction of the lagom extent of variation for a poikilosis component ( Figure 1). The spheres represent a cross-section of a cyclic system which can be visualized as tori ( Vihinen, 2020a). For simplicity and visualization purposes, we concentrate on the cross-sections, the effect on the tori can be understood from this perspective by considering longitudinal effects and interactions.

Variation zones are dynamic: both the positioning of the zone and the extent of variation are variable. They depend on the situation of the system, as well as on intrinsic, external, environmental and other conditions. In Figure 1A the large circle indicates the all the possible variations within one level, the smaller shape indicates the dynamic lagom range of variation in one situation ( Figure 1A). The magnitude of effective variation *E* can be formulated as *E = V – R,* where *V* means variation and *R* the sum of TCM factors and processes ( Vihinen, 2020a).

TCMs limit and reduce consequences of variations to lagom extent in normal situations. The location and extent of lagom varies depending on the situation and condition. Lagom extent of heterogeneity is cost effective and facilitates adequate responses. A system within lagom extent of poikilosis does not need any regulation.

Non-lagom variation at one level can affect connected levels: molecules, processes, pathways, cells, tissues, species and others ( Figure 1E). When variation is excessive, some or many connected levels are affected, and may enter to non-lagom level ( Figure 1F). As the levels are connected to further levels, effects of extensive variations can spread widely. However, even when there is an effect on connected levels, the effect is often so small that the consequences are within lagom for that level and the non-lagom extent is restricted to the original level or just to some additional connected levels. In such a case system-wide effects are limited.

When there is a smaller variation, the system returns to lagom level relatively quickly and there are no major consequences. Larger deviations, such as those causing diseases, may damage, impair or reduce the functionality and adaptability of the system or the entire organism. In the most severe conditions (diseases at organism level), a domino-like spread of non-lagom variation extents affect new levels. The effects and extent vary markedly between diseases and between individuals who have the same disease. In the most extreme case, non-lagom effects continue to spread to new levels finally causing death.

### PLTR model of regulation

Variation appears in practically all biological systems, for an extended list see ( Vihinen, 2020a). As an example, only some 15–25% of genes in the genome are expressed in substantial extent in a given cell at a certain time. Only the so-called housekeeping genes have quite constant expression in any condition. Well-known examples of tightly regulated systems are human blood glucose level and body temperature. Even these processes display some heterogeneity. Systems previously considered as homogeneous have in close inspection been found to show heterogeneity, such as single-cell studies of homogeneous cells and tissues ( Evans *et al*., 2018; Qian *et al*., 2017).

**Figure 3.**
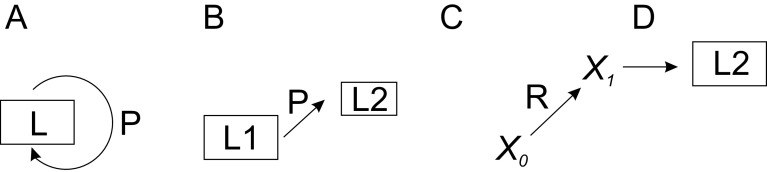
PLTR model of regulation. A. TARAR mechanisms restrict effects of perturbation P and maintain the lagom L state of the system. B. Larger perturbation causes the system to enter from one lagom level L1 to another lagom level L2. C. Regulator R modifies entity *X _0_* to another functional state *X _1_*, after which D) the system establishes new lagom L2. Note that L2 in B and D are different. In A, B and D regulation is in lagom maintenance mode, in C in reguland regulation mode.

A novel generic perturbation-lagom-TCM-regulator (PLTR) model is presented for biological regulation. The model combines the effects of perturbation and lagom in a system with countermeasures and possible regulators. This model can be applied to any biological system and process. There are three modes of regulation, two of which are lagom-related. In the first scenario, lagom is maintained, both intrinsic (passive) and active TARAR countermeasures can be involved ( Figure 3A). In the second mode, there is a shift from one lagom to another ( Figure 3B). The third mode is called reguland regulation, where the regulated entity is a target of regulatory shift, which is often irreversible or requires action of another regulator to return to original state ( Figure 3C). After the shift, the system enters lagom maintenance mode, but at new lagom extent ( Figure 3D).

Regulated systems can be depicted in general form as


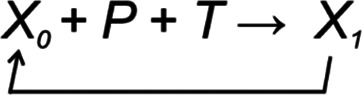

(1)
where *X _0_* is the biological property or feature in the system and *X _1_* is the property after one cycle of regulation. Then, *X _1_* becomes new *X _0_* and the regulation continues. *P* is perturbation of the system, while *T* indicates TARAR countermeasures. *X _0_* can be called reguland ( Connant and Ashby, 1970), analogous to measurand in measurements.

*P* and *T* jointly contribute to the next state of the reguland *X.* The values of *P* and *T* can be positive or negative. Their combined effect defines the new state of the system. As far as *T* keeps the *X* within lagom extent, there is no change to the system. All five types of TCMs can be involved depending on the reguland and the process(es) it is involved in. This mode of regulation is energetically favorable as the robustness of the process restricts the outcome. TCMs functional in a process can be activated or repressed by regulatory processes.

When the perturbation is larger, the system enters to new lagom extent ( Figure 3B). From the point of regulation, the system then continues in the first mode of lagom maintenance. The consequences of even larger, non-lagom, perturbations are discussed below.

Reguland regulation can be described as

$$X_{0}\overset{R}{\to }X_{1}$$ (2)

where *R* indicates regulator that modifies the reguland *X _0_* so that it enters to new state *X _1_*, where it will remain unless another regulator *R _2_* modifies it. This mode of regulation enters the process to a new state as in protein phosphorylation, effects of which can be removed by dephosphorylation by phosphatase activity of regulator *R _2_. R* itself can be regulated; it can be activated by several processes in various situations.

Changes introduced by *R* vary widely depending on the type of process and the type of the regulated entity. Post translational modifications, such as proteolysis, are involved in the activation of many proteins, like proteases ( Stroud *et al*., 1977). Other types of introduced changes are e.g., conformational alterations, as the reorientation of lobes in protein kinases ( Amatya *et al*., 2019), or changes in the structure of a RNA thermometer ( Narberhaus *et al*., 2006). Allostery, change in a site further away from the actual functional site ( Wodak *et al*., 2019), is a further example. Regulatory alterations can be actuated by internal or external mediators, environmental changes, chemical, biochemical, mechanical, physical, cellular, sensory, genetic or epigenetic factors, drugs, hormones, growth factors and others.

Although the formula for lagom maintenance (1) is reminiscent of negative feedback regulation, it is not limited to this type of regulation. Regulatory networks have been investigated extensively in relation to transcription factors and gene expression networks where various types of network motifs have been identified, see e.g. Alon (2007). The motifs include single input modules, negative and positive auto-regulation, feedforward loops (FFLs), multi-output FFLs and dense overlapping regulons, however there is not a consensus for the types of motifs and their definitions. The network motifs and regulatory modes are not limited to transcription processes. Regulation can even be more complex and contain several regulators and regulands. Reguland regulation is a type of feedforward regulation.

Negative feedback regulation, the regulatory mode in the traditional models of physiological regulation, is not cost effective. Regulation of a feature requires a sensor or sensors and a system to control the regulated property. At best, the most effective negative feedback circuits reduce the variation with the fourth root of the number of signalling events (number of control molecules) ( Lestas *et al*., 2010). The number of needed controllers thus grows very rapidly. For example, reduction to one fifth of the uncontrolled heterogeneity demands the minimum of 625 (5 ^4^) -times overproduction of controllers in comparison to the reguland. Already the reduction of heterogeneity to half demands ≥16 times higher number of sensors. Many regulatory systems are complex, and each regulated entity would require their sensors and controllers in large quantities. All regulatory systems have their maximum capacity, once it is reached the system enters either to new lagom level or to non-lagom extent.

### Scenarios and mechanisms of regulation

The regulatory modes and their combinations can explain all kinds of regulatory systems at any level in a biological system. These include, but are not limited to, physiological regulation, gene expression regulation, regulation of signaling pathways, metabolism, ecosystem food webs and ecosystem self-organization. In the following, some widely studied regulatory systems are described with the PLTR model.

Regulation mechanisms can be simple or complex. Control of one process can affect many other processes as systems form networks. Similarly, one system may be regulated by several other processes which again can be regulated by several systems. Factor *X* can be a regulator of another system and it can be regulated by other systems. Many types of network motifs are known ( Alon, 2007). Non-lagom extent of heterogeneity of an entity can affect the lagom and regulation at connected levels.

*lac* operon in *Escherichia coli* is a textbook example of a regulatory system. In the absence of lactose, the *lac* repressor inhibits the expression of the lactose utilization genes from an operon by binding to an operator region in front of the coding genes. Binding of RNA polymerase to the promoter is aided by the cAMP-bound catabolite activator protein (CAP). The expression of repressor is constitutive, unless a co-inducer binds to the repressor. The repressor inhibits transcription by blocking binding of RNA polymerase to the operator of the operon. It can only be removed when co-inducer allolactose binds and inactivates the repressor. As another step of lactose regulation, when glucose, the major carbon source, is available, CAP, which is essential for the lactose utilization gene expression, prevents lactose transport into the cell.

The PLTR model explains the *lac* operon regulation as follows. In the absence of lactose, the system is at a lagom maintenance mode and stays there as long as no lactose is available. The binding of allolactose and inactivation of the repressor is a reguland regulation step where the repressor conformation is changed. The system enters to new state where genes are expressed from the operon. The new lagom is maintained by the lagom maintenance mode. The removal of allolactose reactivates the repressor in another reguland regulation step. CAP regulation is explained by the model as follows. When glucose is available, CAP is inactivated in a lagom maintenance mode and thus preventing lactose permease expression and consequent transport of lactose. The two regulatory mechanisms together regulate *lac* operon, the first one controlling lactose utilization gene expression and the other transport of lactose and allolactose into cells.

At lagom state a *status quo* persists in a system and there is no need for regulation. The system is relaxed and active, energy consuming actions do not occur. Lagom emerges from the combined outcome of TCMs and the complexity of the systems. When *X _1_* is smaller or higher than lagom, intrinsic TCM mechanisms restrict the change and can return the system back to lagom extent of heterogeneity. Attenuation mechanisms are the major TCMs involved in this mode of regulation. Robustness, which originates from the complex organization of the system, buffers and makes systems slow to change their state (systemic inertia) and thereby effectively attenuates small/medium changes and perturbations.

Many enzymes display saturation kinetics - their hyperbolic Michaelis-Menten curves show a long asymptotic tail. Even extensive reduction of the activity may not have a major effect on the flux through the process ( Hartl *et al*., 1985) since even the reduced activity is of lagom extent. In some diseases patients have a severe phenotype only when majority, even 95%, of the normal activity is lost ( Vihinen, 2021a). As many enzyme activities in cells are close to saturation ( Meiklejohn and Hatl, 2002), even a substantially reduced activity is still at lagom extent and does not have a major impact or phenotype. In systems formed of enzymes, such as the pentose phosphate pathway, the flux can be reduced to 15% and in the tricarboxylic cycle to 19% of normal activity without significant effect on optimal growth of *Escherichia coli* ( Edwards and Palsson, 2000). These systems are very robust and the lagom state is maintained in a very wide activity range.

Once the perturbation cannot be controlled by the intrinsic TCMs, adaptive tolerance, repair and/or resistance mechanisms are activated. This type of regulation consumes energy. Depending on the extent of regulation and perturbation, the system returns to the original lagom extent or enters to new lagom. When at the new lagom state, attenuation mechanisms keep that extent.

Chaperones are ever-present in cells, but their expression can also be induced by perturbations. They act as folding chaperones and assist proteins to fold correctly. Repair mechanisms actively correct effects of perturbations. Many types of suppression mechanisms are activated by genetic or protein variants ( Vihinen, 2021a). Rewiring of pathways is relatively common and provides robustness for cells and organisms ( Dueber *et al*., 2004).

Regulator can be external or intrinsic within the system. It either increases or decreases the activity of the factor and can be called different names depending on the system and function, including regulator, activator, enhancer, inducer, inhibitor, insulator, repressor, silencer, and allosteric regulator. In addition to the natural regulators, there can be external factors, such as toxins, addiction-causing substances, drugs and environmental, population and ecosystem changes. Regulator-mediated control is active and consumes energy, however the consumption is small as one regulator can regulate many regulands. TCM mechanisms are active also in this form of regulation, in fact they cannot be switched off.

Emergent processes are common in biology, although often ignored. Whenever the system outcome cannot be derived from the output of independent factors, it is likely that the system has emergent properties. In the case of regulation, robustness is an example of an emergent property ( Kitano, 2004; Masel and Siegal, 2009). It originates from the joint contribution of regulatory processes, molecules, networks and lagom states. System resilience effectively restricts changes to the state of a system and defines the lagom state.

Maintenance of lagom and perturbation control have a range of responses, whereas regulator control is largely binary. Activation or inactivation can be permanent or reversible. Proteolytic activation of many proteases is an example of irreversible form of control, it lasts until the activated protein is degraded, since there is no mechanism to attach inactivating propeptide back to the protein. Certain functions, such as cellular signalling, demand for fast switching on or off. Protein phosphorylation and dephosphorylation are examples of reversible post translational modifications in signalling molecules with immediate effect on system activity.

Physiological regulation is complex, and the regulatory systems are composed of several regulated processes. Processes like glucose level regulation require the concerted action of many factors. Hundreds of genes have been implicated in diabetes type 2 ( Rani *et al*., 2017), although the actual function and role is known just for a few. The regulatory factors can be regulated by other regulators and TCMs forming a large and complicated network. However, the action and regulation of the components can be described by the lagom maintenance and reguland regulation modes in the PLTR model. This has important implications for modelling of biological processes.

### Non-lagom extent of heterogeneity

Regulatory systems are effective, but have maximum capacity, and when the capacity is exceeded, excessive perturbations force the system to leave lagom state. Depending on the excess of the perturbation, the system then enters either to another lagom state, which is stabilized by TCMs, or has non-lagom extent that cannot be controlled. Further perturbation of a system at non-lagom state increases the extent of the property in an uncontrolled way and can cause a disease. Depending on the system, the effects and consequent phenotypes of non-lagom extent vary widely. Non-lagom extent of heterogeneity does not automatically mean that the system and organism are out of control. This situation may be tolerated to a certain extent.

Non-lagom variation can affect connected levels and unless the effects on these levels are controlled by regulatory systems within them, the effect can spread further. The presence of lagom and TCMs in each level effectively restrict and limit the spread of non-lagom heterogeneity to additional levels. However, each of these levels have their maximum capacity.

The most severe diseases and conditions are systemic and perturb many levels. Extreme and uncontrolled variation eventually leads to death. Poikilosis-aware definition states that *death is caused by excessive multilevel variations that irreversibly collapse vital processes and functions and spread to become system-wide* ( Vihinen, 2020a).

### Set point conceptions are not compatible with PLTR control

Homeostasis ( Bernard, 1865; Cannon, 1929) has been considered as one of the cornerstones of modern biology ( Michael, 2007), although it has flaws and there are inconsistencies with experimental data. Therefore, numerous updated or improved theories have been presented in an effort to fit theory with reality, see e.g., Ramsay and Woods (2014). Here a short introduction is provided to these theories.

Allodynamic regulation. The authors claim that at least some physiological changes related to behavioral states could indicate active inhibition of set-point-based regulation ( Berntson and Cacioppo, 2000). Heterostasis is defined as “establishment of a new steady state by exogeneous (pharmacologic) stimulation of adaptive mechanisms through the development and maintenance of dormant defensive tissue reactions” ( Selye, 1973). Set points can be actively changed due to the presence of pathogens. Heterostasis is a wider concept than homeostasis.

Homeodynamics is an extension of homeokinetics ( Yates, 2008). Homeokinetics is homeostasis controlled by dynamic regulation ( Soodak and Iberall, 1978). Homeorhesis is dynamic adaptation to the priorities of the state of the organism ( Bauman and Currie, 1980; Waddington, 1957). Teleorhesis was considered as an alternative name. Homeoreusis is defined as active defense of changing parameters. It allows parameters to change during time ( Nicolaidis, 2011). Poikilostasis means multiple homeostatic states during a lifetime ( Kuenzel *et al*., 1999). Predictive homeostasis is feedforward, anticipatory homeostasis considering circadian and seasonal time. Predictive homeostasis aims to adjust the system before it is expected to escape outside the allowed range ( Moore-Ede, 1986). In reactive homeostasis, feedback restores a system after an environmental challenge ( Moore-Ede, 1986). Reactive scope model is an extension of allostasis and combines predictive and reactive homeostasis with homeostatic overload and homeostatic failure ( Romero *et al*., 2009). Rheostasis is graduated quantitative regulation ( Mrosovsky, 1990). Teleophoresis is similar to homeorhesis, i.e., dynamic adaptation ( Chilliard *et al*., 1998).

Allostasis ( Sterling and Eyer, 1988) is the most widely applied alternative theory. The concepts of homeostasis, allostasis and others are vague and fuzzy ( O'Leary and Wyllie, 2011; Ramsay and Woods, 2014) and it is not even clear whether there are differences between the different theories ( Carpenter, 2004; Day, 2005). The idea of homeostasis was crystallized by Cannon as “wisdom of the body”, but what is this wisdom and how does it work has never been described in detail ( Cannon, 1929).

The key principles of homeostasis and allostasis ( Ramsay and Woods, 2014) are discussed here in relation to poikilosis. Homeostasis was based on the concept of a static ideal state, a “set point”, to which the system is actively returned by negative feedback regulatory loops after any change or perturbation. Set point has subsequently been replaced by “adjustable set point”, an idea that has also been adapted to allostasis. If adjustable set point existed, it would be extremely costly to maintain. Biological control systems are rather inefficient, even in optimal feedback control the ratio of controlling molecules increases in the quartic power along with increased regulation ( Lestas *et al*., 2010). Thus, claims for homeostasis, allostasis and others to be cost effective do not hold. Homeostasis would spend a substantial part of the body’s energy on control. This is not energetically feasible nor is it supported by expression data for control molecules. In the PLTR model there is no set point, instead attenuation and active processes restrict the variability in the system and thereby define lagom extent of poikilosis.

Learned anticipated responses are a key tenet in allostasis and more recently also proponents of homeostasis (it was not originally part of homeostasis) present responses to be controlled and coordinated centrally, by the brain. However, such a central command center does not exist, neither is there necessary signalling for it. This idea would also limit physiological regulation to organisms with substantially large and evolved brains. Centralized regulation simultaneously within cellular compartments is not possible, thereby regulation is local and distributed ( O'Leary and Wyllie, 2011) as PLTR model indicates.

Homeostasis is a tempting theory as it is simple, however, it does not explain reality, and despite it was introduced more than 100 years ago and it has not been clearly and fully described. There are many vague aspects about homeostasis and features that do not match with biological reality. On the contrary, the PLTR model provides a clear mechanistic explanation for all types of biological regulation, not just for physiological regulation.

### PLTR model in medicine

The PLTR model has important connotations for medicine. As mentioned above, diseases are due to excessive variation that spreads to many levels. TCMs restrict poikilosis in normal situations and to some extent even in many non-normal conditions.

Lagom is the normal situation of a biological system, thus any treatments and processes that work towards keeping and retaining lagom poikilosis would be beneficial. As curative treatment, typically targeted towards the main cause of the disease, is not always possible, it would be beneficial to prevent non-lagom effects from spreading. Palliative care aims to optimize quality of life and mitigate suffering of patients, while curative treatment targets the root cause of the disease. Curative care is available only in a fraction of diseases. Some of the diseases with the largest numbers of patients receiving palliative care involve various forms of cancers, chronic respiratory diseases, cardiovascular diseases and diabetes.

The concept of TARAR reconstitution as a therapeutic option was recently introduced ( Vihinen, 2020b). The goal in this therapy is to increase the system robustness and resilience by strengthening TCMs and activating processes that restrict the spread of non-lagom poikilosis to additional levels. It could help to reconstitute and recover the body back to lagom level of poikilosis in as many levels as possible. Once successful, this approach will restrict the total burden of non-lagom extents substantially and prevent many effects on connected levels.

Another approach to take benefit of TARAR reconstitution would be to treat those non-lagom effects and consequences for which there are established treatments available. This could even mean that the primary non-lagom effect is not treated at all. Instead, the burden of non-lagom effects is reduced by treating other level(s) that then could reduce the overall effect to the body. To work, this approach for treatment has to be based on understanding the connected levels and disease mechanisms to choose correct target(s) and treatment(s). This approach could be beneficial in many diseases for which there is currently no curative treatment, and which have substantial impact on the wellbeing of individuals. From the regulatory point of view, the goal is to reconstitute systems to lagom extent in as many affected levels as possible. This principle applies also to comorbidities, where the reconstitution therapy could have a large overall effect when spreading of the non-lagom extent of heterogeneity is reduced.

Immune reconstitution therapy is already used to treat various conditions with impaired immunity including hematopoietic stem cell transplantation in primary immunodeficiencies ( Notaragelo and Pai, 2019), treatment of HIV-infection ( Corbeau and Reynes, 2011), multiple sclerosis ( Lünemann *et al*., 2020) and hematologic malignancies ( van Tilburg *et al.*, 2011).

## Discussion

The new model connects the theory of poikilosis to biological regulation. Previous models of regulation have been applicable only to certain specific types of regulation: homeostasis for physiological regulation, gene expression regulation for molecular biology, etc. Common regulatory motifs have been detected, but a generic model of biological regulation has been missing. The PLTR model facilitates explanation of any biological regulatory system. Homeostasis, allostasis and others are based on negative feedback regulation, which is a mechanism among many others. PLTR is compatible with any mode and mechanism of regulation. Regulation has been discussed here in relation to biology, but since poikilosis also appears in other areas ( Vihinen, 2020a) it is likely that the principle of lagom maintenance and reguland regulation also applies to other fields.

Three regulatory scenarios were identified. In the simplest form, the maintenance of lagom, the intrinsic properties of the system attenuate effects of perturbations and changes. This mechanism works at systemic level and does not demand energy consuming interventions. Somewhat larger perturbations and changes require additional active TCMs. In the second mode of regulation, perturbation causes the system to shift from one lagom state to another. For fast and active control of biological systems there is the third mode of regulation where a regulator modifies a reguland that shifts from one state to another.

The PLTR model provides a framework for explaining any kind of biological system in any organism and at any level. The author is not aware of other regulatory models that would be compatible with poikilosis. Central to the model is the idea of lagom. PLTR regulation is cost effective and facilitates targeted and specific regulation. Even very complex regulatory processes can be broken down to lagom maintenance and reguland regulatory steps.

Consideration of the PLTR model and principles of poikilosis will allow applications to a wide range of biological systems. Regulatory motifs identified in transcription regulation and other regulatory concepts can be explained and elaborated with the PLTR model. Implementation of these models and others in systems biology will assist more reliable and accurate predictions and simulations; however, to facilitate these calculations, poikilosis has to be considered in the experimental studies which the models are based on. It will be necessary to develop experimental approaches and algorithms that can handle poikilosis, perturbation, lagom, TCMs and regulators simultaneously.

Stress was among the topics that allostasis was claimed to explain better than homeostasis ( Sterling and Eyer, 1988). Allostatic load was explained as the wear and tear on the body and accumulation due to repeated or chronic stress. According to the PLTR model, stress, or any other kind of perturbation, does not require special treatment. Stress and the allostatic load can be explained as accumulated multilevel non-lagom effects.

The new model for regulation will also contribute to increased reproducibility in science once the relevant factors in the investigated process are considered. When processes are described in detail, including poikilosis and regulation, experiments and interpretations of obtained results will be more reliable and reproducible.

## Data availability

No data are associated with this article.
