# Supplementary material for: Generic model for biological regulation
Source: F1000Res. 2022 Aug 26;11:419. Originally published 2022 Apr 13. [Version 2] doi: 10.12688/f1000research.110944.2 (PMC9468631; doi:10.12688/f1000research.110944.2)
Supplement: Peer review history

# Reviewer report: Reviewer response for version 2

**DOI:** 10.5256/f1000research.137801.r148670

**Published:** 2022-09-12

**Authors:** Rita Casadio

**Affiliations:** 1 Biocomputing Group, CIG, Interdepartmental Center «Luigi Galvani» for Integrated Studies of Bioinformatics, Biophysics and Biocomplexity, University of Bologna, Bologna, Italy

Author's comments to my observations clarify my issue.

Is the topic of the opinion article discussed accurately in the context of the current literature?

Yes

Are arguments sufficiently supported by evidence from the published literature?

Partly

Are all factual statements correct and adequately supported by citations?

Partly

Are the conclusions drawn balanced and justified on the basis of the presented arguments?

Yes

Reviewer Expertise:

Computational Biology and Biophysics

I confirm that I have read this submission and believe that I have an appropriate level of expertise to confirm that it is of an acceptable scientific standard.

# Reviewer report: Reviewer response for version 2

**DOI:** 10.5256/f1000research.137801.r148669

**Published:** 2022-09-01

**Authors:** Nishanth Ulhas Nair

**Affiliations:** 1 Center for Cancer Research, National Cancer Institute, National Institutes of Health, Bethesda, USA

The author has addressed my comments.

 There is a typo (see the word "Definition") in the newly added Figure 1 which needs to be corrected.

Is the topic of the opinion article discussed accurately in the context of the current literature?

Yes

Are arguments sufficiently supported by evidence from the published literature?

Partly

Are all factual statements correct and adequately supported by citations?

Partly

Are the conclusions drawn balanced and justified on the basis of the presented arguments?

Yes

Reviewer Expertise:

Computational biology, cancer research

I confirm that I have read this submission and believe that I have an appropriate level of expertise to confirm that it is of an acceptable scientific standard.

# Reviewer report: Reviewer response for version 1

**DOI:** 10.5256/f1000research.122607.r145901

**Published:** 2022-08-18

**Authors:** Nishanth Ulhas Nair

**Affiliations:** 1 Center for Cancer Research, National Cancer Institute, National Institutes of Health, Bethesda, USA

The author presents a very theoretical framework on biological systems in this opinion piece. The paper may be interesting for some people working on theoretical biology but it is currently unclear if anyone from experimental biology or computational biology would be interested in it given the lack of specific biological findings. The author must be commended for thinking deeply and abstractly on a high level for this project. I have a few specific concerns.

1. Almost all the prior work on Poikilosis seems to be done by the same author as this paper (mostly self-citations). Are there no other authors who work on this field?
2. There are a lot of definitions for so many different terminologies. However, it was hard to understand the key insight of this work. What is novel and why is this interesting? Since there are so many new definitions, I would suggest including an overview figure connecting all the various terminologies. At present, the paper is hard to understand/follow on how all of this connects.
3. Is there any practical utility to this theoretical framework? Can you provide a clear example?
4. Poikilosis is defined as a new unifying biological theory according to which inherent pervasive variation is a normal state for all biological systems ranging from subatomic particles to the biosphere. Is there experimental evidence for this theory? What does ‘normal state’ mean?
5. How does this theory apply in the case of cancer cells when there is uncontrolled proliferation?
6. There are a few statements made in the paper which are stated like facts but are more likely to be opinions. For example, the statement “The goal of life is survival of the individual and species” is made without any citation. How do we know what the goal of life is? Please search the manuscript throughout to ensure claims are either cited or written by adding caveats like ‘likely’, ‘probably’ etc.

Is the topic of the opinion article discussed accurately in the context of the current literature?

Yes

Are arguments sufficiently supported by evidence from the published literature?

Partly

Are all factual statements correct and adequately supported by citations?

Partly

Are the conclusions drawn balanced and justified on the basis of the presented arguments?

Yes

Reviewer Expertise:

Computational biology, cancer research

I confirm that I have read this submission and believe that I have an appropriate level of expertise to confirm that it is of an acceptable scientific standard, however I have significant reservations, as outlined above.

# Response

**Published:** 2022-08-22

**Authors:** Mauno Vihinen

**Affiliations:** Lund University, Sweden

*The author presents a very theoretical framework on biological systems in this opinion piece. The paper may be interesting for some people working on theoretical biology but it is currently unclear if anyone from experimental biology or computational biology would be interested in it given the lack of specific biological findings. The author must be commended for thinking deeply and abstractly on a high level for this project. I have a few specific concerns.*

 RESPONSE: Thank you very much for the thorough review and pertinent comments.

 The theory of poikilosis and PLTR model are intended to be implemented into practice. Consideration of inherent and pervasive heterogeneity will allow reliable measurements, observations and interpretations of various biological issues.

*1. Almost all the prior work on Poikilosis seems to be done by the same author as this paper (mostly self-citations). Are there no other authors who work on this field?*

 RESPONSE: The concept of poikilosis was published 2 years ago and there have not yet been others working on the topic. Since the new theory demands for novel approach and thinking, it requires some time to be adapted and such works have not yet been published. As discussed in my previous articles and in here, there is plenty of prior work that supports poikilosis.

*2. There are a lot of definitions for so many different terminologies. However, it was hard to understand the key insight of this work. What is novel and why is this interesting? Since there are so many new definitions, I would suggest including an overview figure connecting all the various terminologies. At present, the paper is hard to understand/follow on how all of this connects.*

 RESPONSE: I have added a new figure to explain the central concepts. My initial idea was to avoid redundancy with my prior publications, but now I see a need for clarifying the key concepts also in here. There are just three major concepts (poikilosis, lagom, TARAR countermeasures) from which the entire theory is derived.

*3. Is there any practical utility to this theoretical framework? Can you provide a clear example?*

 RESPONSE: If we are talking about poikilosis and not of PLTR model, the theory provides a framework for more accurate description of basically any process and phenomenon. The differences in results in comparison to current paradigm may be minor in some instances and substantial for others. One practical example is homeostasis. As discussed in the article, several authors have indicated numerous problems with this widely used concept. Poikilosis and PLTR model provide a more reliable and realistic description of biological regulation, for details see the text.

*4. Poikilosis is defined as a new unifying biological theory according to which inherent pervasive variation is a normal state for all biological systems ranging from subatomic particles to the biosphere. Is there experimental evidence for this theory? What does ‘normal state’ mean?*

 RESPONSE: I provide tens of examples of poikilosis in PMID: 32913639, the cited articles describe natural variation and heterogeneity in a wide spectrum of molecules, processes and systems. Heterogeneity is a normal state for any system.

*5.  How does this theory apply in the case of cancer cells when there is uncontrolled proliferation?*

 RESPONSE: Cancer is a complex and complicated disease, which could be considered from several poikilosis points of view.  Cancer cells have lost regulation that controls their growth, they are thus in non-lagom level. Several gene variants and effects are required for cancer to emerge. Together, they bring cells, tissues, organs and finally entire organism to several non-lagom levels, which once the effects are large enough, spread to additional levels and lead to uncontrolled proliferation. This leads eventually to further spreading of cancer and its effects to additional levels and can lead to systemic collapse and death.

*6. There are a few statements made in the paper which are stated like facts but are more likely to be opinions. For example, the statement “The goal of life is survival of the individual and species” is made without any citation. How do we know what the goal of life is? Please search the manuscript throughout to ensure claims are either cited or written by adding caveats like ‘likely’, ‘probably’ etc.*

 RESPONSE: Thank you for the suggestion. The goal of life was discussed earlier in PMID:32913639, I have now added a reference to it. To avoid excessive self-citations, I did not always add a reference to my previous work when I considered that it was clear from the context. I have now added references to such instances.

# Reviewer report: Reviewer response for version 1

**DOI:** 10.5256/f1000research.122607.r137027

**Published:** 2022-05-30

**Authors:** Rita Casadio

**Affiliations:** 1 Biocomputing Group, CIG, Interdepartmental Center «Luigi Galvani» for Integrated Studies of Bioinformatics, Biophysics and Biocomplexity, University of Bologna, Bologna, Italy

1.  Introduction: Is the inherent pervasive variation of a state different in the different cell types? If so, how can we measure this variability? Considering also the previous paper from the same author in FASEB Bio Advances 2021, 3: 611-625. ^1^
2. Poikilosis and lagom : If depends on lagom, do we have to think of a cell steady state?  Which is the difference? In a steady state, fluctuations may drive the system to another state (Irreversible Thermodynamics). Why not address the problem in the framework of thermodynamics?
3. TARAR counter-measures: to which extent the system (an open thermodynamical one) is independent from the context (other cells in the same tissue, organ, ..) and from the environment (nutrients)?
4. PLTR model of regulation: An interesting systemic description of biological regulation. Can this be modelled with different sets of kinetic equations in order to simulate system behavior and evolution? If yes, we are back to a thermos-kinetic framework, with the inclusion of molecular biology details that routinely we describe as isolated subsystems.
5. Set point conceptions are not compatible with PLTR control: Possibly a scheme will help readers to focus on the relevance of the new model compared to previous ones.
6. A very interesting view of how non-normal situations may lead to disorders and death. Elsewhere one would say that the system cannot control entropy production any longer. Will the new model be more amenable to measurements and predictions?

Is the topic of the opinion article discussed accurately in the context of the current literature?

Yes

Are arguments sufficiently supported by evidence from the published literature?

Partly

Are all factual statements correct and adequately supported by citations?

Partly

Are the conclusions drawn balanced and justified on the basis of the presented arguments?

Yes

Reviewer Expertise:

Computational Biology and Biophysics

I confirm that I have read this submission and believe that I have an appropriate level of expertise to confirm that it is of an acceptable scientific standard, however I have significant reservations, as outlined above.

## References

[1] : Measuring and interpreting pervasive heterogeneity, poikilosis. *FASEB Bioadv* .2021;3(8) : 10.1096/fba.2021-00015 611-625 10.1096/fba.2021-00015 34377957PMC8332472

# Response

**Published:** 2022-06-01

**Authors:** Mauno Vihinen

**Affiliations:** Lund University, Sweden

I am very thankful for the comments and questions, they all rise important aspects which are clarified in the following.

1. *"Introduction: Is the inherent pervasive variation of a state different in the different cell types?" *
**RESPONSE**: Yes, it is different even between individual cells of the same type. Poikilosis emerges in every system as heterogeneity. Naturally, variation e.g. within similar cells in a tissue is smaller than between cells of different types. However, there is heterogeneity that has to be considered in accurate descriptions and models.
*"If so, how can we measure this variability? Considering also the previous paper from the same author in FASEB Bio Advances 2021, 3: 611-625.1"*
**RESPONSE**: It depends on the system. The article the Reviewer mentions elaborates the issue. Systematic, controlled experiments can be used to chart the extent of heterogeneity, however, may not fully represent the biological system. It is apparent that more experiments and experiments with more variables than currently customary are needed to chart the extent of heterogeneity and lagom.
2. *"Poikilosis and lagom : If depends on lagom, do we have to think of a cell steady state?  Which is the difference? In a steady state, fluctuations may drive the system to another state (Irreversible Thermodynamics). Why not address the problem in the framework of thermodynamics?" *
**RESPONSE**: We can consider lagom to represent a kind of steady state in a cell or other system. When the system is within lagom, no action is needed or in the case of somewhat larger deviation, countermeasures return the level back to lagom. Lagom is not a single state, instead an interval of lagom heterogeneity. Within a lagom state, the system will not drift to another lagom state as there is no driving force. For moving lagom to another state there has to be either large enough perturbation, major change in system component(s) or e.g. a reguland regulatory step.
  Whether thermodynamic principles and approaches could be used to describe and predict such situations is a topic of further studies.
3. *"TARAR counter-measures: to which extent the system (an open thermodynamical one) is independent from the context (other cells in the same tissue, organ, ..) and from the environment (nutrients)?" *
**RESPONSE**: We have to start to look at this from the concept of level. Levels are any molecules, processes, reactions, networks, populations etc. They are or can be highly connected to other levels. How they are connected and to which other levels and how environmental conditions affect the system is dependent on the level and situation. TARAR countermeasures are specific or generic and restrict and reduce the variation of the level. Many countermeasures are also regulated. They form highly connected and complex networks.
4. *"PLTR model of regulation: An interesting systemic description of biological regulation. Can this be modelled with different sets of kinetic equations in order to simulate system behavior and evolution? If yes, we are back to a thermos-kinetic framework, with the inclusion of molecular biology details that routinely we describe as isolated subsystems."*
**RESPONSE**: The goal of this paper is to describe the new model of regulation. The implementation of the model to practice will require extensive research in each individual level and system. It will be essential to make systematic perturbations and to introduce other changes to the system. New types of approaches, or modifications of existing ones, are needed e.g. to take the interval of lagom into account. Current models are not well suited for this. Kinetic models, when modified to take e.g. lagom and TARAR countermeasures into account, might be applicable in some situations; however, they are not generic and applicable to all regulated systems and processes ranging from subatomic level to the biosphere.
5. *"Set point conceptions are not compatible with PLTR control: Possibly a scheme will help readers to focus on the relevance of the new model compared to previous ones."*
**RESPONSE**: The previous conceptions are mentioned only briefly as they are not compatible with poikilosis and the new model of regulation. They have been developed to different degrees of maturity and as such do not warrant even comparison between them. Homeostasis has been discussed in a large number of papers, some other concepts have been introduced just in a single study, others are somewhere in between. All the models introduced after homeostasis try to fix some flaws of homeostasis.
6. *"A very interesting view of how non-normal situations may lead to disorders and death. Elsewhere one would say that the system cannot control entropy production any longer. Will the new model be more amenable to measurements and predictions?"*
**RESPONSE**: The reviewer has it right, the entropy increases during disease and cannot be controlled in the most extreme cases. The new model facilitates more reliable description of regulated systems than achievable based on current theories. Instead of just a single regulation mechanism in homeostasis (negative feedback regulation) all types of regulation are acceptable and thereby in line with experimental evidence. To achieve more reliable predictions, new methods or implementations are needed. These methods have to handle with the interval of lagom extent, cover level interactions, perturbations and other changes in the system. Further, they need to consider the three states of regulation and develop mathematical formulations for them and their transitions.
